# Time-restricted feeding causes irreversible metabolic disorders and gut microbiota shift in pediatric mice

**DOI:** 10.1038/s41390-018-0156-z

**Published:** 2018-08-28

**Authors:** Dandan Hu, Yilei Mao, Gang Xu, Wenjun Liao, Jinjun Ren, Huayu Yang, Jun Yang, Lejia Sun, Hongyu Chen, Wenda Wang, Yanan Wang, Xinting Sang, Xin Lu, Hongbing Zhang, Shouxian Zhong

**Affiliations:** 1 Department of Surgery, Peking Union Medical College Hospital, Beijing, China; 2 Department of General Surgery, Second Affiliated Hospital of Nanchang University, Nanchang, Jiangxi China; 3 Department of General Surgery, Henan Cancer Hospital, Zhengzhou, Henan China; 4 Institute of Basic Medical Sciences, Chinese Academy of Medical Sciences, Beijing, China

## Abstract

**Background:**

Time-restricted feeding regimen (TRF), that is, no food consumption for 14–16 h during the light phase per day, attenuates the fattening traits and metabolic disorders in adults. This study aims to further investigate whether TRF would be protective against similar nutritional challenges in juvenile mice.

**Methods:**

Mice in the experimental group were treated with TRF during the first 4 weeks (considered to be the childhood phase of mice) before switching to ad libitum (AD) feeding pattern as adults; the control group with all subjects sticks to AD mode. Body weight was monitored, and serum biochemistry, sexual maturity, immune function, and gut microbiota were assessed at a certain timing.

**Results:**

Mice treated with TRF during the childhood period (from weaning age) but went through AD feeding pattern as adults demonstrated the tendency of higher body weight, higher levels of serum glucose, shrunken Langerhans islets, fatty liver disease, thickening of aortic walls, delayed sexual development, increased proportion of T regulatory cells, and unhealthy gut microbiota.

**Conclusion:**

Childhood TRF causes pleiotropic adverse effects, including severe irreversible metabolic disorders, depressed immune function, and retarded puberty. Microbiota set the stage for TRF to employ downstream reactions on the above changes.

## Introduction

The worldwide prevalence of metabolic syndrome has significantly increased in recent decades, causing great threat to both adult and pediatric populations. Overweight, impaired glucose homeostasis, hypertension, and dyslipidemia, often followed by cardiovascular disease, diabetes mellitus, neurodegenerative diseases, and fertility problems, have jointly become a major burden to all societies,^1^ and could even lead to delayed diagnosis of severe diseases, and increase the risk of cancerous diseases.^2^ Exercise, bariatric surgeries, and dietary regimens including caloric restriction, intermittent energy restriction, and time-restricted feeding (TRF) are effective interventions for obesity and metabolic syndrome in adults.^3^ Furthermore, fasting can reduce obesity and obesity-related diseases but can also provide benefit to the neurological system and lifespan.^4,5^

Previous studies demonstrated that TRF is superior to surgical interventions and other dietary patterns, because it is not invasive and does not lead to unbearable hunger, which is critical for daily use.^6^ Our previous study using an ischemic–reperfusion model showed that TRF is protective against liver impairment (unpublished data). Furthermore, the eating habits of our hunter–gatherer ancestors largely resembled TRF, rather than the three meals a day in present-day society.^7^ TRF was reported to relieve or reverse conditions associated with obesity, diabetes mellitus type 2, hyperlipidemia, fatty liver disease, and inflammation.^8^ Over the past 5 years, various studies have investigated the mechanism of TRF. The ingestion of nutrients induces the acute expression of multiple clock genes,^9^ which strengthen circadian rhythm,^10^ optimize cell functions,^8^ and lower the incidence of cancerous diseases, by manipulating gene expression profiles and mitochondrial electron transmitting complex^11^ that have an impact on biochemical pathways.^8^ This also has an impact on mitochondrial function, DNA repair, autophagy, stem cell renewal, the restoration of blood glucose homeostasis,^12^ and conversion to the ketogenic pathway, which consumes fat, causing weight loss, and providing a more diverse gut flora.^13^

Although the physiology of children is distinct from adults, all previous TRF studies have focused on adults. Therefore, the consequences of TRF in children are unknown. Considering the unique physiologic features of children, we were extremely cautious regarding the extrapolation of the positive effects of TRF observed in adults. The current study provides evidence for the use of TRF in healthcare policy and practice in the pediatric population. Further studies should translate the detrimental physiological findings observed in mice to humans, which will provide information for policy makers regarding the appropriate diet for the younger generation.

## Materials and methods

### Animals

All animal experiments were performed in accordance with the Institutional Animal Care and Use Committee guidelines of the Salk Institute. Experimental animal care was conducted with the permission of the Animal Welfare Committee of Peking Union Medical College Hospital (permission no. XHDW-2015-1098). Kunming male mice (Beijing Vital River Laboratory Animal Technology Co., Ltd. (4 Yangshan Road, Beijing, China)) at 3 weeks of age (weaning age) were entrained to a 12:12 light–dark cycle. Intervention started after acclimatization with normal chow food available ad libitum (AD) for 1 week. Week 0: mice were 4 weeks old (3 weeks of breastfeeding + 1-week normal chow diet for acclimatization). Week 4: mice were 8 weeks old and their feeding pattern was changed to the experimental method. Week 4 was considered when juvenile mice developed into adult mice. Week 8: mice were 12 weeks old, and the main outcome data were collected and analyzed.

### Feeding schedule and diets

At Week 0, mice were randomly assigned to two feeding regimens. In the experimental group, mice followed the TRF pattern, while mice in the control group had access to food AD. Under TRF, mice were allowed access to food between zeitgeber time 13 (ZT 13) (ZT 0: lights on; ZT 13: 1 h after lights off) and ZT 21 (3 h before lights on).

Caloric intake was unrestricted in either regimen. Both regimens used normal chow (GB14924.3-2010 Standard: 29% protein, 13% fat, and 58% carbohydrate) (by reference: LabDiet-5010). Food was replenished at ZT 13 each evening to assure an adequate amount of food was available for each cage.

*Definition of experimental and control groups*: TRF–AD (TA) mice followed TRF during the first 4 weeks (5–8 weeks after birth, after acclimatization), which was considered the juvenile period. After 4 weeks, juvenile developed into adult mice and were allowed food AD. AD-AD (AA) mice were designated as the control group and all individuals were fed AD. The TRF-TRF (TT) group was forced to fast during a 16-h period daily throughout the experiment.

### Food consumption and weight

The amount of food consumed for each cage was weighed every day at ZT 21, and body weight was monitored weekly.

### Serum biochemistry

Blood was obtained at ZT 21 (beginning of the fasting period for TRF). Enzyme linked immunosorbent assay was used to measure the concentration of serum insulin, triglyceride (TG), total cholesterol (TC), low-density lipoprotein (LDL), high-density lipoprotein (HDL), leptin, ghrelin, peptide YY (PYY), glucagon-like peptide 1 (GLP-1), gonadotropin-releasing hormone (GnRH), follicle-stimulating hormone (FSH), luteinizing hormone (LH), androgen, and estrogen (E). Blood glucose levels were measured with a OneTouch Ultra glucose meter at ZT 13 and ZT 21.

### Histology study

Heart, liver, and pancreas were isolated from mice at ZT 21 (Week 8) and fixed in formaldehyde solution. Specimens were sectioned and stained with a standard hematoxylin–eosin staining (H&E) method.

### Flow cytometry analysis

The quantity of regulatory T cells (Tregs) was determined by flow cytometry as follows: (1) whole blood was collected at ZT 21 in the presence of an anticoagulant; (2) cells were stimulated and incubated at 37 °C for 30 min; (3) cells were washed, and permeabilized; (4) cells were resuspended in Staining Buffer at a final concentration of 5–10 × 10^6^ cells/mL; (5) Phosflow antibodies were added—fluorescein isothiocyanate-conjugated anti-CD4, allophycocyanin-conjugated anti-CD25, phycoerythrin-conjugated anti-Foxp3—and incubated for 60 min in the dark; (6) cells were washed, centrifuged, and the supernatant was decanted; (7) cells were resuspended prior to flow cytometric analysis; and (7) C6 flow cytometer (BD Accuri) was used to identify the composition of lymphocytes.

### Metagenomic DNA extraction and 16S rRNA sequencing

Three mice (from different cages) from each feeding group were sacrificed at ZT 21 (Week 8). Individual mouse ceca were isolated from the rectum and flash frozen. Ceca were then resuspended and digested before lysis. DNA from the lysate was extracted, precipitated, washed, and resuspended. 16S rRNA gene sequence tags, corresponding to the hypervariable V1–V3 region, were generated using the 454 pyrosequencing platform. Operational taxonomic unit (OTU)-based classification was used to generate data profiles. To acquire a general understanding of the microbiota, α and β diversity analyses were performed, predominantly to show diversity between and within groups.

### Statistical analysis

Data were analyzed by Stata/MP 14.0. All normally distributed data were displayed as the mean ± s.e.m. Comparisons between two groups were performed by the Student’s *t* test. A value of *p* < 0.05 indicated statistical significance.

## Results

### Childhood TRF caused metabolic disorders

**Fig. 1:**
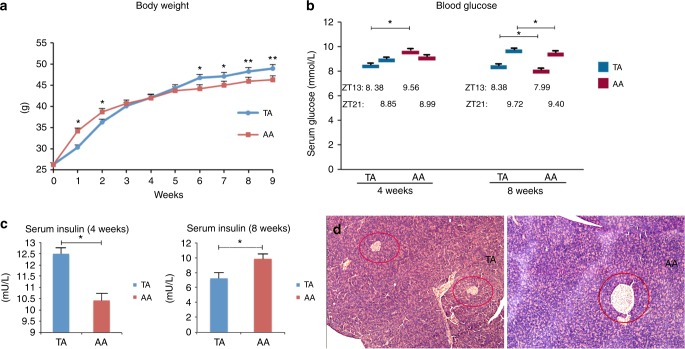
Body weight and glucose metabolism. **a** Weekly measured body weight of time-restricted feeding of juvenile (TA) and ad libitum fed (AA) mice. *n* = 40. **b** Blood glucose levels of both groups at Weeks 4 and 8. Samples harvested at ZT 13 and ZT 21 (before and after feeding in TRF mode). *n*_TA_ = 10, *n*_AA_ = 16. **c** Serum insulin concentration at Weeks 4 and 8. Week 4 (left): *n* = 12, Week 8 (right): *n*_TA_ = 11, *n*_AA_ = 6. **d** H&E-stained histological sections of the pancreas (10 × 10-fold magnification). *p < 0.05; **p < 0.01. TA: left, AA; right, Langerhans islets are circled in red

*Body weight* (Fig. 1a): TA mice demonstrated weight loss under TRF intervention, but were more obese when allowed to eat AD in adulthood. Although the weight of the TA group was generally lower than the AA group for the first 4 weeks, the mean weight of mice in the TA and AA groups were not significantly different (40.08 ± 0.90 vs. 40.71 ± 0.68 g, *p* = 0.30; *n* = 40). The amount of food consumed by mice in both groups were measured, yet no significant difference was revealed at the end of week 4 when comparing TA group to AA group: 5.16 ± 0.20 vs. 5.20 ± 0.18 g/day. However, weights of mice in the TA group increased after week 4 and remained high until the end of the experiment. At Weeks 8 and 14, the mean weights of the TA and AA groups were 47.1 ± 0.82 vs. 45.0 ± 0.93 g (*p* < 0.01), and 48.9 ± 0.68 vs. 46.3 ± 0.73 g (*p* < 0.01), respectively.

*Glucose metabolism*: Before changing the feeding pattern in the TA group (end of Week 4), the mean serum blood glucose levels for the TA and AA groups were 8.38 ± 0.39 and 9.56 ± 0.34 mmol/L at ZT 13 (TA lower than AA, *p* < 0.05), and 8.85 ± 0.16 and 8.99 ± 0.34 mmol/L at ZT 21 (no statistical difference), respectively (Fig. 1b). After the TA group was switched to the AD mode for 4 weeks (Week 8), the opposite trend was observed. TA serum glucose levels reached 8.38 ± 0.20 at ZT 13 and 9.72 ± 0.16 mmol/L at ZT 21. Levels at both timepoints were significantly higher compared with the control group (Z13: 7.99 ± 0.14 mmol/L, ZT 21: 9.40 ± 0.18 mmol/L, *p* < 0.05). At the end of Week 4, serum insulin levels were measured (Fig. 1c). Samples were collected at ZT 21, after the feeding period for TRF mice. The TA group had a higher insulin concentration compared with the AA group (12.494 ± 0.27 vs. 10.420 ± 0.38 mU/L, *p* < 0.05), which was attributed to the feeding pattern TA mice were resigned: blood was collected right after their restricted feeding period. But the reverse was observed at Week 8, where both groups were offered absolutely the same AD regimen. The TA group had a lower insulin concentration compared with the AA group (7.199 ± 0.76 vs. 9.860 ± 0.63 mU/L, *p* < 0.05). The sizes of Langerhans islets were small in TA mice (Fig. 1d), which might explain the lower level of insulin in this group. The mean radius of TA pancreas islets was 30% of the size of the pancreas islets in the AA group (*n* = 16).

**Fig. 2:**
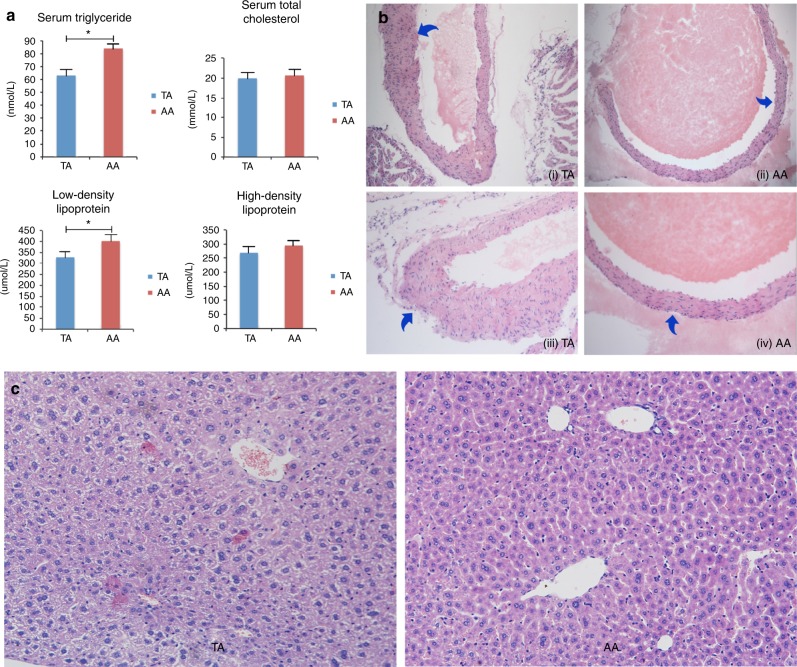
Serum lipid concentrations and histological study of aortic root and liver. **a** Serum lipid levels at the end of Week 8. *n*_TA_ = 13, *n*_AA_ = 6. **b** H&E-stained aortic roots from TA and AA mice under 10 × 10 (i and ii) and 10 × 20 (iii and iv) magnification. (i) and (iii): TA group; (ii) and (iv): AA group. **c** Representative H&E-stained histological sections of liver: left, TA group; right, AA group (10 × 20 magnification)

*Lipid metabolism*: Concentrations of serum triglyceride, total cholesterol, low-density lipoprotein, and high-density lipoprotein in the TA and AA groups were (Fig. 2a): 62.92 ± 6.48 vs. 83.86 ± 4.34 nmol/L (*p* < 0.05), 19.95 ± 1.51 vs. 20.64 ± 2.05 mmol/L (*p* = 0.39), 327.87 ± 23.42 vs. 401.56 ± 35.61 μmol/L (*p* < 0.05), and 253.04 ± 20.89 vs. 295.21 ± 14.79 μmol/L (*p* = 0.13). Aortas of TA mice were rigid with hyaline thickening and proliferation of walls (Fig. 2b). However, no classic signs of atherosclerosis (fibrous plagues within the intima, ulceration, hemorrhage, or calcification) were observed. TA mice tended to have fatty liver disease. Livers were harvested from 16 mice per group at Week 8, and H&E-stained histological sections revealed fatty changes in the TA group. No focal liver cell necrosis, infiltration of neutrophils, or cirrhosis was observed (Fig. 2c).

### Changes of digestive hormones and peptides

**Fig. 3:**
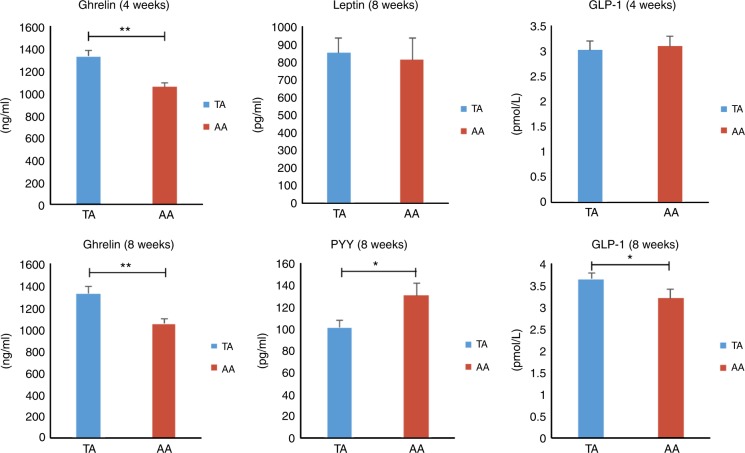
Serum levels of digestive hormones and peptide at Weeks 4 and 8. Digestive hormone and peptide concentrations at Weeks 4 and 8 (*n* = 12). *p < 0.05; **p < 0.01

(1) *Leptin*: At Week 8, serum leptin concentrations in the TA group were 849.48 ± 86.55 pg/mL, which were slightly higher than in the AA group, 808.33 ± 124.99 pg/mL (*p* = 0.263). (2) *Ghrelin*: At Week 4, ghrelin in the experimental group was significantly higher (1325 ± 60 ng/L) than in the control group (1053 ± 39 ng/L) (*p* < 0.01). At Week 8, similar levels were observed in both groups (TA: 1325 ± 70 ng/L vs. control: 1050 ± 44 ng/L; *p* < 0.01). (3) *PYY*: The PYY concentration was significantly lower at Week 8 in the TA group (100.55 ± 6.70 pg/mL) compared with the AA group (129.84 ± 11.58 pg/mL; *p* < 0.05). (4) *GLP-1*: No significant difference was observed between groups at Week 4, but GLP-1 levels increased to 3.65 ± 0.15 pmol/L in mice with early TRF. Mice in the AA group showed a gradual increase in GLP-1 to 3.21 ± 0.22 pmol/L (*p* < 0.05) 4 weeks later (Fig. 3).

### TA mice showed retarded sexual maturity and depressed immune function

**Fig. 4:**
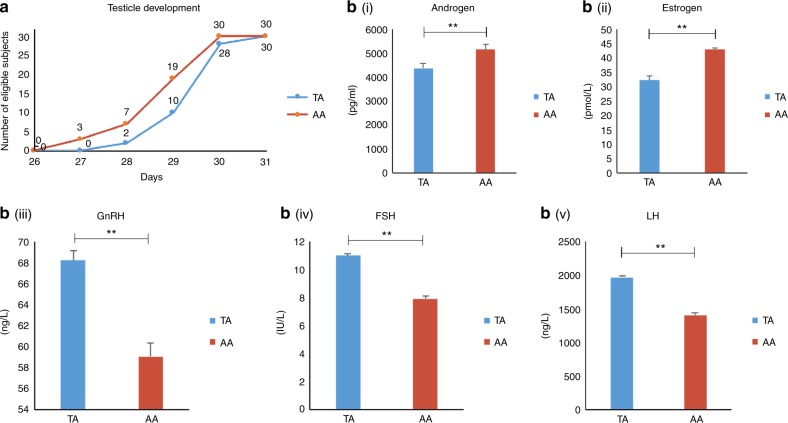
Sexual development of TA and AA mice. **a** Number of mice in each group with “testicle development,” documented daily from day 27 onwards (*n* = 30). TA: time-restricted feeding as a juvenile, AA: ad libitum fed. **b** Serum sex hormone levels at Week 4 (*n* = 6). **p < 0.01

*Sexual development*: On Day 27, three AD mice showed enlarged testicles. As the testicles are elliptical, we measured the largest diameter and defined “enlarged testicles” as ≥8 mm. Using this standard, we counted the number of mice under TRF or AD with “testicle development” (Fig. 4a). By Day 31, all mice had mature testicles. TA mice were more likely to have developmental retardation compared with the AA group. Blood sampling to measure sex hormones was performed on Day 32, because this timepoint provided the best evidence of sexual development based on changes in testicle size. Data were highly consistent: high levels of GnRH, FSH, LH, and low levels of downstream hormones including androgen and estrogen were observed in the TA group (all *p* < 0.01). This indicated that TA mice showed a lack of development, whereas AA mice matured normally.

*Treg cells*: At Week 8, we used fresh peripheral blood samples to measure the percentage of CD4+CD25+Foxp3+ Tregs in all lymphocytes. The mean Treg percentage (Treg%) for the TA group was 6.78 ± 1.41%, and 2.69 ± 0.31% for the AA group (*n* = 6, *p* < 0.01).

### Ecologic imbalance of gut microbiota interpreted multiple negative effects

**Fig. 5:**
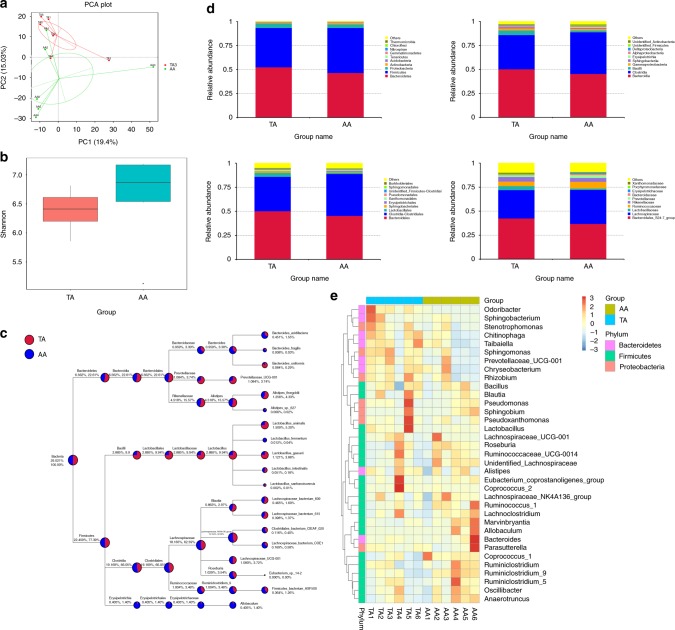
Analysis of gut microbiota at Week 8. **a** Principal component analysis (PCA) of all subjects. **b** α Diversity of groups by Shannon analysis (*p* = 0.24). **c** Taxa-tree of bacteria species by groups. **d** Top 10 bacteria in abundance under phylum, class, order, and family level of TA and AA groups. **e** Taxa-heatmap under genus level of each subject being analyzed (*n* = 6)

PCA analysis demonstrated that different feeding intervention has a prolonged influence on gut microbiota even after they were both treated with AD for 4 weeks. Comparing with AA group, TA group was low in α diversity (*p* = 0.24) (Fig. 5a, b). Subsequent taxon analysis upon different levels demonstrated specific bacteria were different in abundance by groups (Fig. 5c–e).

### Continued TRF in adulthood failed to reverse the existing impairments

**Fig. 6:**
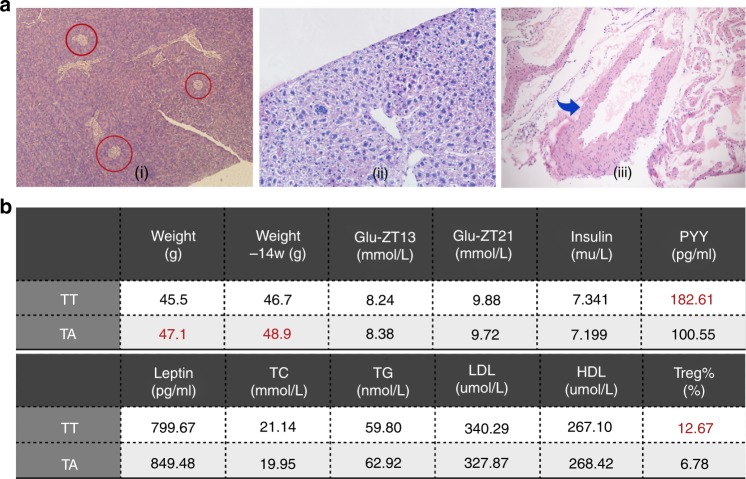
Outcome indicators of mice under TRF both as children and adults. **a** H&E-stained histological sections of organs and tissues from TT mice. (i) Pancreas, red circles indicate Langerhans islets (10 × 10 magnification), (ii) liver (10 × 10 magnification), and (iii) aorta root, blue arrow indicates vessel wall (10 × 4 magnification). **b** Values of TT compared with TA at Week 8 (except weight at Week 14). Statistical significance is indicated in red. TT time-restricted feeding as a juvenile and adult

Continuing TRF throughout adulthood did not reverse existing impairments of mice who underwent TRF in the first 4 weeks. (i) Langerhans islets in the pancreas were smaller with a mean radius similar to the TA group. (ii) Fatty changes were more severe compared with the TA group. (iii) Rigid and thickened vessel wall of the aortic root (Fig. 6a). Four outcome values showed significant differences: the mean weight of TT mice was lower (45.5 ± 1.01 g) than in the TA group (47.1 ± 0.82 g; *n* = 40; *p* < 0.05). The mean weight of the TT group at Week 14 was lower (46.7 ± 0.75 g) compared with the TA group (48.9 ± 0.68 g; *n* = 10; *p* < 0.05). The mean weight of the PYY group was higher (182.61 ± 11.70 pg/mL) than in the TA group (100.55 ± 6.70 pg/mL; *n*=11; *p* < 0.01). The Treg% in the TT group was almost twofold higher than in the TA group (12.67 ± 2.23% vs. 6.78 ± 1.41%; *n* = 6; *p* < 0.01). There were no significant differences in the other eight variables (glucose level, insulin, leptin, TC, TG, LDL, HDL) between the TT and TA groups (Fig. 6b).

## Discussion

Accumulated evidence has demonstrated that optimizing dietary structure and feeding schedule, including TRF, can prevent or even reverse the unfavorable results of metabolic syndrome. However, we reported that TRF caused severe dysmetabolism in a juvenile murine model, instead of the presumed improvements in general health conditions. TRF mice in childhood had high body weights and blood glucose levels in adulthood, as well as a suppressed immune system, and delayed sexual maturation.^14^ At the end of Week 4 in this study, before the feeding pattern of the TA group was changed, mice of TA and AA groups consumed similar amounts of food and there was no difference in body weight between groups. At Week 8, the mean body weight of the TA group was higher than the AA group, although TA mice ate slightly less (not statistically significant). We repeated the weight measurements at Week 10 and the trend remained unchanged. A similar finding was observed for serum glucose concentrations, as TA mice developed hyperglycemia. At Week 4 (ZT 13), mice under AD pattern had higher glucose levels compared with those treated with TRF, as they had access to food AD, but the difference was reduced at ZT 21. However, at Week 8, when both groups were under AD feeding, TA mice developed hyperglycemia. As previous reports have fully revealed that TRF led to better outcomes both with normal and high-fat diet, we used normal chow to investigate specifically into the impact of feeding timing. Yet, the unexpected increase in weight and blood glucose level of the TA group made us curious as to whether TRF in childhood might adversely lead to future metabolic syndrome. Thus, we investigated other possible manifestations at Week 8. Insulin resistance did not occur; instead, the mean insulin level of the TA group was lower than the control group, and pancreas specimens observed under a microscope demonstrated that the size of Langerhans islets was smaller in the TA group compared with the AA group in accordance with the low insulin levels. This contradicts the idea of type 2 diabetes, in which the level of insulin would increase, along with the number and volume of Langerhans islets.^15^ Instead, type 1 diabetes mellitus (T1DM) characterized by insulin deficiency caused by pancreatic β-cell loss leading to hyperglycemia might be a better explanation, but nonetheless we need further investigations of the presence of related autoantibodies, proteomics, insulin dependence, and insulitis. Because the feeding pattern was the only intervention, this suggests that the level of digestive-related hormones and peptides might be different between groups. Changes in leptin were observed in overweight mice;^16^ however, our data showed no statistical differences in leptin concentrations between the TA and AA groups. We hypothesized that a low concentration of leptin in TA mice was confounded by the low absolute value of adipocytes that secrete leptin. Ghrelin stimulates the appetite, which explains the high level of ghrelin in the TA group at Weeks 4 and 8. PYY is a satiety-promoting peptide,^17^ and it was present at a very low level in the TA group. The three indicators mentioned above implied that the desire to eat was generally stronger in TA mice than in AA mice. However, GLP-1 is also a member of the nutrient-induced satiety pathway, but there was no difference in its levels between all groups at Week 4, and it was higher in the TA group at Week 8. It should be noted that GLP-1 stimulates pancreatic β cell repair and regeneration, as well as inhibiting cell apoptosis and improving glucose responsiveness.^18^ Taking together the reduced size of the Langerhans islets and low level of insulin, it is reasonable to assume that GLP-1 was increased by a compensatory mechanism.

The tendency of irreversible depressed immune function and sexual development retardation as well as dysmetabolism of lipids were observed in mice fed time-restrictively before puberty. T1DM is an autoimmune disease that develops mainly during childhood or adolescence. Its etiology is thought to involve the T cell-mediated destruction of β cells, and etiology-associated manifestations include decreased GLP-1 levels, low proportion of Tregs, and compositional changes in gut microbiota.^19^ At Week 8, we analyzed the number of peripheral regulatory T cells by flow cytometry. High numbers of Tregs indicated a fragile immune system and an increased risk of cancer, while interaction with perturbed metabolism and inflammatory states further deteriorate the general health condition. The CD4+CD25+Foxp3+ Treg/lymphocyte ratio was higher in the TA group compared with the AA group, indicating suppressed immune conditions. However, taxon analysis of the gut microbiota in the TA group was not in accord with autoimmune-induced T1DM, namely the trends of Ruminococcus, Bacteroides, and the Prevotella genera were opposite to reported studies. Our data indicated that TA mice were born with normal glucose metabolic capacity, but this became impaired by environmental factors during development. Regarding lipid metabolism, low serum levels of TC, TG, HDL, and LDL in the TA group indicated the malabsorption of lipids. Interestingly, specimens of the liver and root of aorta showed that hypolipidemic TA mice had fatty liver, along with rigid, thickening, and abnormal proliferation of the aortic walls. TRF was previously shown to alleviate oxidative stress and lower the risk of cardiovascular disease;^20^ therefore, the above results suggest that TRF causes devastating metabolic impairments in the young in contrast to adults through a network that has not been fully elucidated. A previous study showed that TRF had a positive influence on fertility.^21^ Considering the feasibility and sample size, we did not design experiments for the complete assessment of sexual ability including the number of births per delivery, survival rate, and interval between pregnancies. Nevertheless, some mice fed AD in the AA group showed enlarged testicles ahead of the other group. We found that the TA group had delayed puberty compared with the AA group. To confirm this observation, we collected blood samples and measured sex-related hormones. Estrogen and androgen (downstream sex hormones) of AA mice were higher than in the AA group. In contrast, TA mice had higher levels of upstream sex hormones including GnRH, LH, and FSH than in the AA group. These results indicate that when AA mice became sexually mature, TA mice were still under active development. Because TRF can improve both general health conditions and relieve diabetes mellitus,^22,23^ we investigated the above impairments induced by childhood TRF could be reversed. However, there were no differences in serum glucose, insulin, lipids, and pathology of the liver, pancreas, and aorta in mice fed TRF from childhood to adulthood (TT group) compared with the TA group. However, TT mice had a lower weight, higher PYY level, and extremely high ratio of Tregs, almost twice as much as in the TA group. These findings indicate that TT mice have a low body weight, reduced immune system, and no improvement in impaired metabolic functions, which demonstrated that damage caused by TRF at an early age is irreversible.

Microbiota allows crosstalk between diet and health impairments, and our own findings in microbiota test provided further proof for our above-reported outcomes. The human body communicates with the exterior environment through complex mechanisms. The diverse and dynamic gut flora constitutes a vital part of this machinery with profound effects on a broad spectrum of organs and tissues.^24^ Microbiota have a direct impact on the metabolic status of their hosts by regulating the energy yield from food and modulating metabolic pathways, influencing metabolic disease progression.^25^ Complex carbohydrates and plant polysaccharides that cannot be degraded by human enzymes are fermented in the colon to yield end products that function as inflammatory modulators, vasodilators, and regulators of gut motility,^26^ and PYY GLP-1 levels to influence the host’s satiety.^27^ Gut microbiota also confer a survival advantage against bacterial infection.^28^ The microbiome plays a fundamental role in the induction, training, and function of the host immune system, and it is associated with many immune diseases including Sjögren syndrome, systemic lupus erythematosus, and acquired immune deficiency syndrome.^29^ Certain kinds of gut flora produce proteins that alter immunoreceptors on T cells and natural killer cells to block their cytotoxic activity on tumor cells.^30^ Commensal bacteria have a role in circadian regulation by producing glucocorticoids of intestinal epithelial cells.^31^ Symbiosis between gut microbiota and intestinal epithelial cells requires the integrity of the circadian clock.^32^ Maternal gut flora contributes to postnatal development and the microbiome of young mice.^33^ Studies reported that the microbiota has a critical role in the maturation of the host immune system by remodeling tertiary lymphoid structures such as the lymphatic follicles and cryptopatches,^34^ forming the intestinal barrier by the development of epithelial cells and intestinal angiogenesis, and by balancing T cell differentiation. Indeed, germ-free mice have an immature gut-associated lymphoid tissue and imbalance between T-helper and T-regulatory lymphocytes.^35^ As mentioned above, T1DM involves gut microbiota through the balance between effector T cells and Tregs, but our findings regarding TRF and T1DM indicated that diets had effects on T1DM mediated through a different pathway with a congenital autoimmune onset pattern. In previous studies, germ-free mice were reported to have small Peyer’s patch sizes and reduced numbers of CD4+ T cells and immunoglobulin A-producing plasma cells.^36^ Emerging data suggest that the development of a healthy neural system depends on environmental cues, such as molecular signals from the gut. The microbiome is also associated with anxiety, depression, cognition, and autism spectrum disorder.^37^ The microbiome is a short-term indicator that 3–4 days of accommodation would possibly wipe out all previous variation. However, in our experiment, even though the feces samples were collected in Week 8, when all subjects were fed AD for at least 4 weeks, the TA group yet demonstrated relatively unhealthy gut flora compared with the AA group. While increase in α diversity is considered to be positive indicator of gut ecosystem, TA group showed lower value of α diversity compared to blank controls. Subsequent taxon analysis observed decreases in Firmicutes phylum, Clostridiales order, Ruminococcaceae family, and Lachnospiraceae family among the individuals of TA group, and it was documented that these bacteria contributed to the battle against overweight, inflammatory bowel disease (IBD), and colorectal cancer.^38^ On the contrary, the abundance of Proteobacteria phylum and Gammaproteobacteria class were increased in the TA group, while these bacteria were positively associated with IBD. Therefore, we announce that TRF in childhood is a negative indicator of a healthy gut ecosystem, which, upon this diet, contribute negatively to subsequent host health issues.

This study had some limitations. As a pilot study prior to an investigation of TRF in human children, our results were unexpected. The results of the pathological studies were not scientifically quantified: the pancreas was not sectioned in a standardized pattern to calculate the volume of Langerhans islets, and severity of fatty liver disease was not indicated by systemic evaluation. Metabolic phenotyping was not thoroughly performed, and results were not adequately repeated due to the sample size. The other defect is that the serum level of leptin was not adjusted by fat content via magnetic resonance imaging.^16^

## Conclusion

This is the first study of TRF in childhood. We found that TRF induced irreversible damage in mice involving metabolic disorders, increased cardiovascular risks, suppressed immune function, and delay of sexual development. Microbiota mediated these downstream effects of TRF. These detrimental outcomes and persisting legacy effects indicate that TRF should not be used in children, but further mechanistic investigations are need to fully understand the network among diets, gut microbiota, metabolism, and developmental biology. It is important to remind public health authorities that guidance regarding meal quantity and timing for school-aged children merits consideration.
